# Operative treatment of cystic prolactinomas: a retrospective study

**DOI:** 10.1186/s12902-023-01343-0

**Published:** 2023-05-04

**Authors:** Weijie Su, Kejun He, Yibing Yang, Jiakun Xu, Xixi Li, Hongxing Tang, Jia Yang, Lixuan Yang

**Affiliations:** Neurosurgery Unit, The First Affiliated Hospital, Sun Yat-sen University, Guangzhou, China

**Keywords:** Cystic prolactinomas, Transsphenoidal surgery, Surgical remission, Dopamine agonists, Prolactinomas

## Abstract

**Background:**

The optimal therapeutic approach for cystic prolactinomas remains unclear. This study aimed to evaluate the remission rates of prolactinoma patients after surgical treatment and the risk factors affecting postoperative remission in cystic prolactinoma patients.

**Methods:**

The clinical data were retrospectively compiled from 141 patients with prolactinomas (including 41 cases of cystic prolactinomas, 21 cases of solid microprolactinomas and 79 cases of solid macroprolactinomas) who underwent transsphenoidal surgery (TSS) between April 2013 and October 2021 at the First Affiliated Hospital of Sun Yat-sen University.

**Results:**

Early postoperative remission was achieved in 65.83% (n = 27/41) of cystic prolactinomas, 80.95% (n = 17/21) of solid microprolactinomas and 40.51% (n = 32/79) of solid macroprolactinomas. The mean length of follow up in all patients was 43.95 ± 2.33 months (range: 6-105 months). The follow-up remission rates were 58.54%, 71.43% and 44.30% in cystic, solid micro- and solid macroprolactinomas, respectively. For cystic prolactinomas, the early postoperative remission rates in the patients with preoperative dopamine agonists (DA) treatment were significantly higher than those without preoperative DA treatment (*p =* 0.033), but the difference in the follow-up remission rates between these two groups was not significant (*p =* 0.209). Multivariate stepwise logistic regression analysis indicated that tumor size and preoperative prolactin (PRL) levels < 200 ng/ml were independent predictors for early postoperative remission in cystic prolactinomas.

**Conclusion:**

For cystic prolactinomas, tumor size and preoperative PRL levels were independent predictors of early postoperative remission. Preoperative DA therapy combined with TSS may be more beneficial to cystic prolactinoma patients.

## Background

Prolactinomas represent the most common functional pituitary adenomas (PAs), accounting for approximately 40% of all PAs [1]. Clinical presentations in prolactinomas patients correlate with either hormonal dysfunction along the hypothalamic-pituitary axis (HPA; e.g., menstrual cycle irregularities galactorrhea and infertility in women, decreased libido and impotence in men) or mass effects (e.g., headache or dizziness, visual flied defects and hypopituitarism) [2]. To normalize hyperprolactinemia and reduce tumor volume, Dopamine agonists (DA) serve typically first-line treatment for prolactinomas patients. With DA, 72% of macroprolactinomas and 78% of microprolactinomas patients are successfully treated [3]. Indication for transsphenoidal surgery (TSS) include patient preference, DA resistance or intolerance, progressive visual impairment and pituitary apoplexy during DA therapy. Cystic prolactinomas are considered when 50% of the sellar lesion volume comprised of cyst. However, cystic prolactinomas show poor response to DA due to the absence of dopamine receptors in the cystic component, which was suggested to be treated with surgical resection according to the Pituitary Society Guidelines (2006) [4]. A retrospective study, consisted of 30 cystic prolactinomas, indicated that over 80% of these patients occurred significant cyst reduction after being treated with DA, but half the patients ultimately underwent surgical resection [5]. In a series of 56 consecutive prolactinomas, remission was achieved in 76% of surgically treated cystic prolactinomas [6]. Thus, optimal therapeutic approach for cystic prolactinomas remains controversial.

The purpose of this study was to assess remission rates in a series of consecutive prolactinomas patients who underwent TSS in our centre and further investigate the risk factors related to postoperative remission in cystic prolactinomas patients.

## Materials and methods

### Patient population

A consecutive series of 141 consecutive prolactinoma patients who underwent TSS at the First Affiliated Hospital of Sun Yat-sen University between April 2013 and October 2021 were retrospectively analysed. The diagnosis of prolactinomas was based on the overproduction of PRL, clinical symptoms and post-surgery immunohistochemistry. The consecutive series included 57 male and 84 female patients (aged 15–68 years, mean 34.52 ± 1.03 years). Based on the tumor texture and maximum tumor diameter, prolactinomas were divided into cystic prolactinomas (> 50% cystic tumor component), solid microprolactinomas (< 1 cm) and solid macroprolactinomas (≥ 1 cm). Forty-one patients (29.08%) harbored cystic prolactinomas, 21 patients (14.89%) had solid microprolactinomas, and 79 patients (56.03%) had solid macroprolactinomas. The indications for surgery in these patients were classified as follows: (1) personal preference; (2) intolerance to DA therapy; (3) persistent hyperprolactinemia (PRL level > 20 ng/ml after 6 months of DA therapy [15 mg bromocriptine daily or 2 mg cabergoline weekly] [7]); and (4) inadequate tumor shrinkage (tumor volume shank less than 50%). Due to the high price of cabergoline, which is not included in the local health insurance policy, most prolactinoma patients were initially treated with bromocriptine and few were treated with cabergoline after bromocriptine. Plurihormonal prolactinomas and patients who did not receive surgical resection were excluded from the study. Patients with Rathke’s cyst or craniopharyngioma were also excluded. Tumor invasiveness was determined by magnetic resonance imaging (MRI) according to the Knosp classification [8].

### Follow-up and remission criteria

Follow-up was performed at one week, three months after surgery, and yearly thereafter at the outpatient department. MRI was routinely performed 3 months after surgery to evaluate the extent of surgical resection and then annually to evaluate tumor recurrence.

Preoperative PRL levels were defined as PRL levels after DA treatment and one week prior to the surgery. Early postoperative remission was defined at 1 week after TSS, as a lack of hyperprolactinemia symptoms and PRL levels < 20 ng/ml. Long-term remission was defined as the absence of hyperprolactinemia or DA medication at the latest follow-up before November 2022. The mean length of follow up in all patients was 43.95 ± 2.33 months, ranging from 6 to 105 months.

### Statistical analysis

Data collection and analyses were performed using Statistical Product and Service Solutions (SPSS) 25.0 (IBM, Armonk, NY, USA). The mean ± standard deviation (SD) represents the date in the study. Student’s t-test, chi-square test/Fisher’s exact test and one way ANOVA were used depending on the scaling and distribution of the variables. The Kaplan‒Meier method was applied to construct the follow-up remission curve. Predictors of early postoperative remission after cystic prolactinoma resection were analysed by univariate and multivariate stepwise logistic regression. Statistical significance was considered as a value of p < 0.05.

## Results

### Clinical characteristics

Clinical features of 141 prolactinomas patients are listed in Table 1. The patients with cystic prolactinomas and solid microprolactinomas were significantly younger than those with solid macroprolactinomas (*p* < 0.001, *p =* 0.007, respectively). The difference of mean age between the patients with cystic prolactinomas and the patients with solid macroprolactinomas was not significant (*p =* 0.728*).* The proportion of females who had solid microprolactinomas was significantly higher than those who had solid macroprolactinomas (90.48% vs. 48.10%, *p* < 0.001). The maximum tumor diameter in cystic, micro- and macroprolactinomas were1.86 ± 0.10 cm, 0.77 ± 0.03 cm and 2.55 ± 0.14 cm, respectively. The tumor size in the patients with solid macroprolactinomas were significantly larger than in the patients with cystic prolactinomas and solid microprolactinomas (*p* < 0.001, *p* < 0.001, respectively). The difference of tumor size between cystic prolactinomas and solid microprolactinomas was significant (*p* < 0.001*).* Invasive prolactinomas were defined as knosp grade IIII or IV prolactinomas according to the knosp classification. In total, 8 of 41 (19.51%) patients with cystic prolactinomas and 47 of 79 (56.96%) patients with solid macroprolactinomas had invasive tumors. All the patients with solid microprolactinomas had non-invasive tumors. The invasive rate was found to be significantly higher in solid macroprolactinomas compared with cystic prolactinomas and solid microprolactinomas (*p* < 0.001, *p* < 0.001, respectively). The patients with cystic prolactinomas had a higher risk of invasiveness compared with the patients with solid microprolactinomas (*p* < 0.001). Visual defects were presented in 15 (36.59%), 2 (9.52%) and 26 (32.91%) patients with cystic, solid micro- and macroprolactinomas, respectively. The proportion of visual defects in the patients with cystic prolactinomas and solid macroprolactinomas were significantly higher than those with solid microprolactinomas (*p =* 0.034, *p =* 0.032, respectively). The differences of proportion of headache, amenorrhea or decreased libido among these three groups were not significant. The preoperative PRL levels were found to be significantly higher in the patients with solid macroprolactinomas compared with those with cystic prolactinomas and solid microprolactinomas (*p* = 0.033, *p* = 0.035, respectively). The difference of the preoperative PRL levels between cystic prolactinomas and solid microprolactinomas was not statistically significant (*p* = 0.692). The total resection rate in the patients with cystic, solid micro- and macroprolactinomas were, respectively 87.80%, 100% and 74.68% (*p* = 0.157 (cystic vs. micro), *p* = 0.093 (cystic vs. macro) and *p* = 0.006 (micro- vs. macro)). Persistent hyperprolactinemia served as the most common reasons for surgical resection among the three groups (13/41 (31.71%) of the cystic prolactinomas, 9/21 (42.86%) of the solid microprolactinomas and 27/79 (34.18%) of the solid macroprolactinomas). The differences in indications for surgery among these three groups was not statistically significant. Of 41 patients with cystic prolactinomas, 27 (65.83%) achieved early remission at 1 week after surgery. 17/21 (80.95%) and 32/79 (40.51%) patients with solid micro- and macroprolactinomas had early hyperprolactinemia remission, respectively. The early postoperative remission rates in the patients with solid macroprolactinomas were significantly lower than that in the patients with cystic prolactinomas and solid microprolactinomas (*p* = 0.008, *p* = 0.001, respectively). The difference of the postoperative remission rates between cystic prolactinomas and solid microprolactinomas was not significant (*p* = 0.215). At the latest follow-up, the remission rate in the patients with cystic, solid micro- and macroprolactinomas were, respectively 58.54% (24/41), 71.43% (15/21) and 44.30% (35/79). The follow up remission rate in the patients with solid microprolactinomas was significantly higher than that in those with solid macroprolactinomas (*p* = 0.027). The follow up remission rate in the patients with cystic prolactinomas was not significantly different from those with solid microprolactinomas (*p* = 0.320), neither with solid macroprolactinomas (*p* = 0.139).

**Table 1:** **Clinical features of prolactinomas patients**

Clinical features	Cystic prolactinoma (n = 41)	Solid microprolactinoma (n = 21)	Solid macroprolactinoma (n = 79)	P values cystic vs. micro, cystic vs. macro, micro vs. macro		
**Age (years)**	29.39 ± 1.56	30.48 ± 1.79	38.25 ± 1.47	0.728^*^	< 0.001^*^	0.007^*^
**Sex (%)**						
Female	27 (65.85%)	19 (90.48%)	38 (48.10%)	0.063^†^	0.064※	< 0.001^†^
Male	14 (34.15%)	2 (9.52%)	41 (51.90%)			
**Tumor size (cm)**	1.86 ± 0.10	0.77 ± 0.03	2.55 ± 0.14	< 0.001^*^	< 0.001^*^	< 0.001^*^
**Knosp grades**						
I	7 (17.07%)	20 (95.24%)	12 (15.19%)	< 0.001^†^	< 0.001^†^	< 0.001^†^
II	26 (63.41%)	1 (4.76%)	22 (27.85%)			
III	8 (19.51%)	0 (0%)	23 (29.11%)			
IV	0 (0%)	0 (0%)	22 (27.85%)			
**Clinical symptoms**						
Visual defects (%)	15 (36.59%)	2 (9.52%)	26 (32.91%)	0.034^†^	0.723※	0.032^†^
Headache (%)	12 (29.27%)	6 (28.57%)	28 (36.71%)	0.954※	0.173※	0.555※
Amenorrhea or decreased libido (%)	25 (60.98%)	17 (80.95%)	46 (58.23%)	0.111※	0.771※	0.055※
**Preoperative PRL level (ng/ml)**	350.66 ± 54.24	209.38 ± 27.16	900.36 ± 196.18	0.692^*^	0.033^*^	0.035^*^
**Total resection (%)**	36 (87.80%)	21 (100%)	59 (74.68%)	0.157^†^	0.093※	0.006^†^
**Indication for surgery**						
Patients’ preference	12 (29.27%)	4 (19.05%)	21 (26.58%)	0.766※	0.977※	0.864※
DA-intolerance	5 (12.20%)	3 (14.29%)	11 (13.92%)			
Persistent hyperprolactinemia	13 (31.71%)	9 (42.86%)	27 (34.18%)			
Inadequate tumor shrinkage	11 (26.83%)	5 (23.81%)	20 (25.32%)			
**Early postop remission (%)**	27 (65.83%)	17 (80.95%)	32 (40.51%)	0.215※	0.008※	0.001※
**Follow-up remission (%)**	24 (58.54%)	15 (71.43%)	35 (44.30%)	0.320※	0.139※	0.027※

^*^ One way Anova; ^†^ Fisher’s exact; ※Chi-square test; DA = Dopamine agonists

### Analysis of cystic prolactinomas

**Fig. 1:**
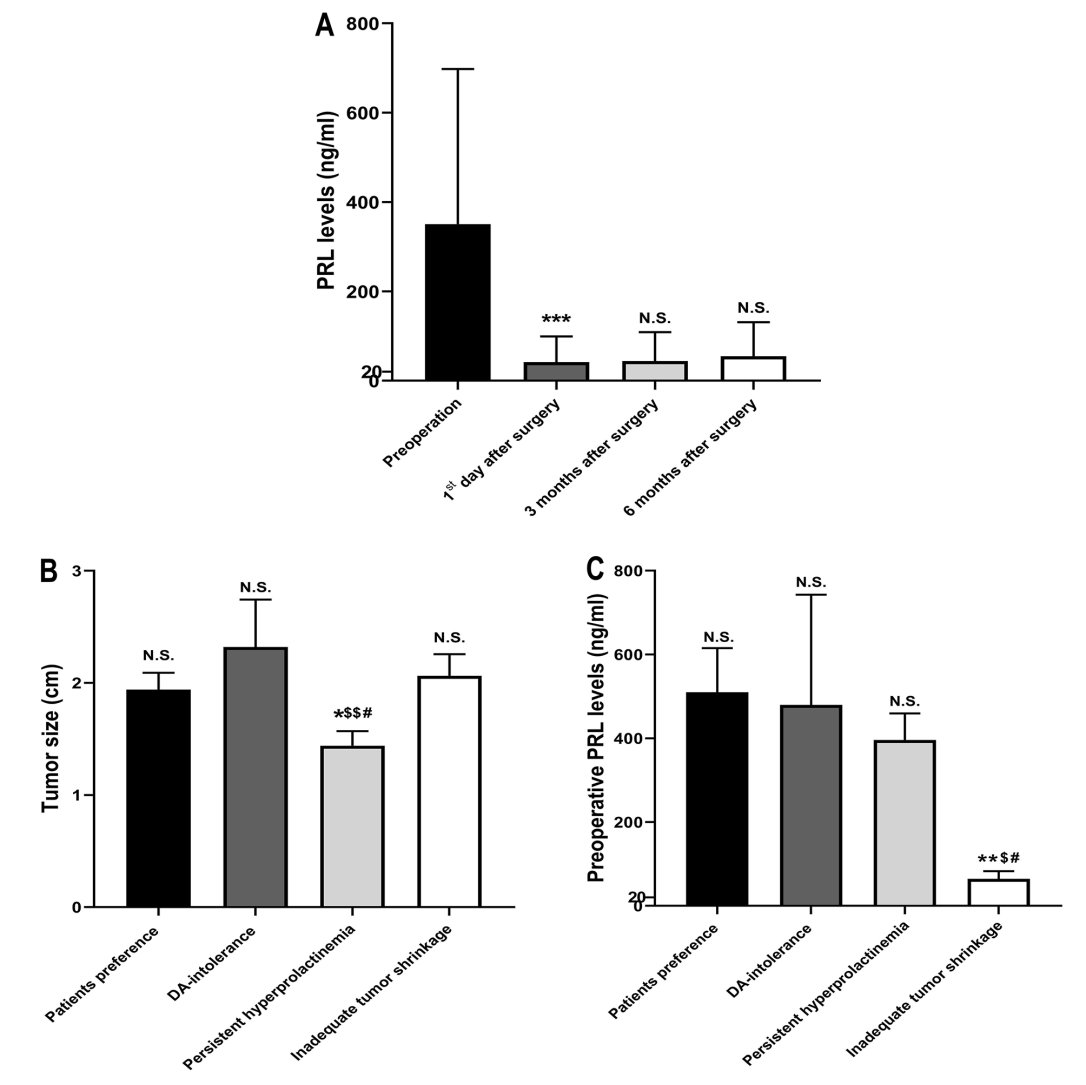
(**A**) Changes in prolactin (PRL) levels in patients with cystic prolactinomas (***p < 0.001). (**B**) Tumor size in cystic prolactinoma patients with different surgical indications (*p < 0.05 vs. patients’ preference; ^$$^p < 0.01 vs. DA-intolerance; ^#^p < 0.05 vs. inadequate tumor shrinkage). Preoperative PRL levels in cystic prolactinoma patients with different surgical indications (**p < 0.01 vs. patients’ preference; ^$^p < 0.01 vs. DA-intolerance; ^#^p < 0.05 vs. persistent hyperprolactinemia)

The mean preoperative PRL levels in the patients with cystic prolactinomas were 350.66 ± 54.24 ng/ml. On the first postoperative day, mean PRL levels declined to 41.76 ± 8.97 ng/ml, which was significantly lower than preoperative PRL levels (*p* < 0.001; Fig. 1A). The mean PRL levels at 3 months and 6 months after surgical resection were 43.93 ± 10.09 ng/ml and 54.68 ± 11.91 ng/ml, respectively. There was no significant difference between these two groups (*p =* 0.074; Fig. 1A). The difference between the mean PRL levels on the 1st day and 3 months after surgery was not statistically significant (*p =* 0.690; Fig. 1A). Among 41 patients with cystic prolactinomas, 13 patients (31.71%), presented with persistent hyperprolactinemia under DA therapy and underwent surgical resection. In total, 12 (29.27%) patients experienced TSS for their own preferences, 11 (26.83%) for inadequate tumor shrinkage and 5 (12.20%) for DA-intolerance, respectively. The mean tumor size in the cystic prolactinomas patients who underwent TSS due to persistent hyperprolactinemia was 1.44 ± 0.13 cm, and in those for their own preferences was 1.94 ± 0.15 cm. In the cystic prolactinomas patients with inadequate tumor shrinkage and DA-intolerance, the mean tumor sizes were respectively 2.06 ± 0.19 cm and 2.32 ± 0.42 cm. Among these four groups, the mean tumor size in the patients with persistent hyperprolactinemia under DA therapy was significantly lower than the other groups (*p =* 0.043 vs. patients’ preferences, *p =* 0.008 vs. DA-intolerance, *p =* 0.015 vs. inadequate tumor shrinkage, respectively; Fig. 1B). The preoperative PRL levels in the cystic prolactinomas patients who underwent surgical removal for inadequate tumor shrinkage were 64.50 ± 18.41 ng/ml, which was significantly lower than the other groups (*p =* 0.001 vs. patients’ preferences (509.96 ± 105.09 ng/ml), *p =* 0.017 vs. DA-intolerance (479.70 ± 263.22 ng/ml), *p =* 0.012 vs. persistent hyperprolactinemia (396.11 ± 63.44 ng/ml); Fig. 1C).

In the patients’ preferences group, 5 of 12 (41.67%) patients had early remission after surgery; 3 of 5 patients (60%) with DA-intolerance achieved early postoperative remission. Early remission was achieved in 10 of 13 (76.92%) patients with persistent hyperprolactinemia and 9 of 11 (81.82%) patients with inadequate tumor shrinkage. The early postoperative remission rates in the patients with preoperative DA treatment were significantly higher than those without preoperative DA treatment (*p =* 0.033; Table 2). The follow-up remission rates in the patients’ preferences and DA-intolerance groups were the same as early remission rates. The follow-up remission rates in the patients with persistent hyperprolactinemia and with inadequate tumor shrinkage were 61.54% (n = 8/13) and 72.73% (n = 8/11), respectively. However, the difference of follow-up remission rates between patients without and with preoperative DA treatment were not statistically significant (*p =* 0.209; Table 2). In addition, the difference in the total resection rates between these two groups were also without statistical significance (*p =* 1.000; Table 2).

**Table 2:** Clinical Characteristics of cystic prolactinomas patients

Features	Without preop- DA treatment		With preop- DA treatment		P values
	Patients’ preference (n = 12)	DA-intolerance (n = 5)	Persistent hyperprolactinemia (n = 13)	Inadequate tumor shrinkage (n = 11)	
**Early postop remission (%)**	5 (41.67%)	3 (60%)	10 (76.92%)	9 (81.82%)	0.033
**Follow-up remission (%)**	5 (41.67%)	3 (60%)	8 (61.54%)	8 (72.73%)	0.209
**Total resection (%)**	10(83.33%)	5 (100%)	11 (84.62%)	10 (90.91%)	1.000

### Factors related to postoperative remission after cystic prolactinomas resection

**Fig. 2:**
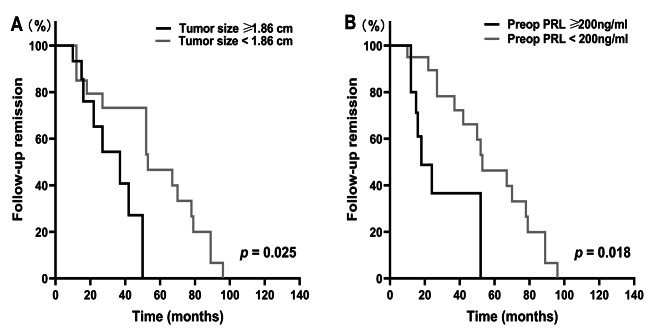
Kaplan–Meier analysis of follow-up remission in 41 patients with cystic prolactinomas according to: (**A**) tumor size and (**B**) preoperative prolactin (PRL) levels

After operation, 27 of 41 (65.83%) patients with cystic prolactinomas achieved early remission. At the latest follow-up, 24 (58.54%) patients with cystic prolactinomas remained remission with a mean follow-up of 32.68 ± 4.19 months (range: 6–96 months). Also, 3 of 27 (11.11%) patients who achieved postoperative early remission had a relapse of hyperprolactinemia. To further investigate clinical factors related to postoperative remission after cystic prolactinomas resection, logistic regression and Kaplan‒Meier analysis were performed. Univariate logistic regression showed that females had higher remission rate (Odds ratio (OR): 0.214, 95% confidence interval (CI): 0.053–0.864, *p* = 0.03; Table 3). Moreover, patients with tumor size ≥ 1.86 cm (mean maximum tumor diameter of cystic prolactinomas patients) had a higher remission rate (OR: 0.136, 95%CI: 0.03–0.615, *p* = 0.01; Table 3). Of the 41 patients with cystic prolactinomas, 20 (48.78%) patients were with a PRL < 200ng/ml preoperatively. Patients with preoperative PRL < 200 ng/ml achieved higher remission rate (OR: 0.160, 95%CI: 0.036–0.717, *p* = 0.017; Table 3). Through multivariate stepwise logistic regression, the present study found that tumor size and preoperative PRL ≥ 200 ng/ml were independent factors affecting postoperative remission (*p* = 0.016, *p* = 0.017, respectively; Table 3). Age, invasiveness, resection degree and resistance to DA therapy were not significant factors related to early remission. Kaplan‒Meier analysis was applied to construct the follow-up remission curve and compare the follow-up periods between two groups. The Kaplan‒Meier analysis indicated that tumor size and preoperative PRL ≥ 200 ng/ml were significant predictors of followed-up remission (*p* = 0.025, *p* = 0.018, respectively; Fig. 2).

**Table 3:** Predictors of early postoperative remission after cystic prolactinomas resection

Predictor	Univariate logistic regression		Multivariate stepwise logistic regression	
	OR (95% CI)	P	OR (95% CI)	P
**Age ≥ 29 years**	2.000(0.500-7.997)	0.327	-	-
**Sex**	0.214(0.053–0.864)	0.030	0.955(0.137–6.656)	0.963
**Tumor size ≥ 1.86 cm**	0.136(0.030–0.615)	0.010	0.072(0.008–0.616)	0.016
**Invasiveness**	0.435(0.090–2.095)	0.299	-	-
**Resection degree**	0.442(0.045–4.387)	0.486	-	-
**DA-resistant**	2.667(0.707–10.052)	0.147	-	-
**Preop PRL ≥ 200 ng/ml**	0.160(0.036–0.717)	0.017	0.081(0.010–0.636)	0.017

DA = Dopamine agonists; OR = Odds ratio; CI = Confidence interval

## Discussion

Prolactinomas are the most common functional PAs and have been recognized to be effectively treated with DA (e.g., bromocriptine and cabergoline) since the 1970s [9]. Binding to the dopamine subtype 2 receptor (D2R) on lactotroph cells, DA can suppress PRL secretion, shrink tumor size and restore gonadal function effectively [10]. For patients with medically resistant prolactinoma, medication intolerance, apoplexy or cerebrospinal fluid fistula, TSS is an option for prolactinomas treatment [11]. Recent studies indicated that TSS may be an alternative first-line treatment to DA in well-selected microprolactinomas patients. In a cohort of 114 microprolactinomas patients, 100 patients (88%) achieved long-term remission through TSS [12]. Another retrospective analysis of 105 microprolactinomas patients who underwent TSS showed that early postoperative remission and long-term remission were 85.7% and 74.3%, respectively [13]. Cystic prolactinomas are a unique type of functional PA, in which the cystic component accounts for more than 50% of the tumor volume. It was reported that the occurrence of cystic prolactinomas is associated with necrosis of the adenoma, spontaneous haemorrhage into the tumor, or combination of the trauma, radiation therapy and use of anticoagulants [14, 15]. Therefore, they are potentially more resistant to DA and surgical treatment was suggested to be the first-line therapeutic approach for patients with large cystic prolactinomas [7, 16, 17]. A previous study enrolled 212 prolactinoma patients reported that after being treated with surgical resection, the overall remission rate at the latest follow-up was 80% in cystic prolactinomas, and 84.8% in microprolactinomas [16]. Another retrospective study indicated that the long-term remission rate in cystic macroprolactinomas patients treated with surgery alone was significantly higher than that in those treated with medication plus surgery (69.2% vs. 33.3%) [17]. In addition, surgical treatment can decrease the pressure on the optic chiasm rapidly due to lesion enlargement while the patient is on medical treatment [18]. However, some studies have demonstrated that DA remains the first-line therapy for cystic prolactinomas without indications for early surgical treatment. In a consecutive series of six cystic prolactinomas treated with DA primarily, half of the patients had remission, and tumor volume shrinkage was achieved in 50% of the patients [19]. Another retrospective study reported that after being treated with DA primarily, over 80% of cystic prolactinoma patients had tumor size reduction (median time to cyst reduction was 24.6 weeks), and 83% of the patients showed biochemical remission [5]. Therefore, the therapeutic approach for cystic prolactinomas remains unclear.

In the present study, the clinical data of 141 patients with surgically treated prolactinomas (including 41 cystic prolactinomas) were retrospectively reviewed. The early remission rates in patients with solid macroprolactinomas were significantly lower than those in patients with cystic prolactinomas and solid microprolactinomas (40.51% vs. 65.83% and 40.51% vs. 80.95%, respectively). The follow-up remission rate in patients with solid macroprolactinomas was significantly lower than that in patients with solid microprolactinomas (44.30% vs. 71.43%) but not significantly different from that in patients with cystic prolactinomas (44.30% vs. 58.54%). For cystic prolactinomas, the mean tumor size in patients who underwent TSS for persistent hyperprolactinemia was significantly lower than that in patients with their own preferences, DA-intolerance and inadequate tumor shrinkage. In addition, the preoperative PRL levels in patients who underwent surgical removal for inadequate tumor shrinkage were significantly lower than those in the other groups. These results indicated that DA is able to reduce tumor size or preoperative PRL levels to a certain extent in cystic prolactinoma patients who are resistant to DA. It has been reported that long-term bromocriptine treatment in prolactionoma patients may cause tumor fibrosis, leading to increased difficulty of gross total resection [7]. A previous study showed that the total resection rate was lower in cystic prolactinoma patients who were treated with DA plus surgery than in those treated with surgery alone [17]. However, our study found that total resection rates in cystic prolactinoma patients with and without preoperative DA treatment were not significantly different. Furthermore, our study also indicated that tumor size and preoperative PRL levels < 200 ng/ml were independent predictors for postoperative remission in cystic prolactinomas using multivariate logistic regression analysis. The early postoperative remission rates in cystic prolactinoma patients with preoperative DA treatment were significantly higher than those without preoperative DA treatment, but the difference in follow-up remission rates between these two groups was not statistically significant. Sughrue et al. demonstrated that preoperative DA treatment was able to improve the resection degree of the tumor and led to better control of the PRL levels [20]. Therefore, our results emphasize the value of preoperative DA treatment in cystic prolactinoma patients for reducing tumor size or preoperative PRL levels to a certain extent. When these lesions are resistant to DA treatment, TSS should be adopted. For those patients without remission after TSS, DA treatment should be performed postoperatively, but its long-term effectiveness warrants further investigation. For patients with progressive visual impairment and pituitary apoplexy, TSS should be the first choice.

This study has inherent limitations; for example, the cohort of cystic prolactinoma patients was not large enough due to the low incidence of cystic prolactinomas. In addition, the study did not analyse cystic prolactinomas who treated with DA primarily due to the in-completed data of these patients. Because cabergoline is not included in the local health insurance policy on the Chinese mainland, most of the enrolled patients were treated with bromocriptine initially in the study and few were treated with cabergoline after bromocriptine. Cystic prolactinomas are commonly large in size [18], and the mean tumor size of cystic prolactinomas in the present study was 1.86 ± 0.10 cm. The analysis of the effect of tumor size on postoperative remission was based on the mean tumor size, which may cause bias.

## Conclusion

In summary, our data indicated that early remission rates after TSS of prolactinomas remained excellent in solid microadenomas and cystic prolactinomas. Tumor size and preoperative PRL levels were independent factors related to remission after cystic prolactinomas resection. For cystic prolactinoma, preoperative DA therapy combined with TSS may be more beneficial to patients.

## Data Availability

All data generated or analyzed during this study and supporting our findings are included and can be found in the manuscript. The raw data can be provided by corresponding author on reasonable request.

## Abbreviations

TSS: Transsphenoidal surgery
DA: Dopamine agonists
PRL: Prolactin
PAs: Pituitary adenomas
HPA: Hypothalamic-pituitary axis
MRI: Magnetic resonance imaging
SPSS: Statistical Product and Service Solutions
SD: Standard deviation
OR: Odds ratio
CI: Confidence interval
D2R: Dopamine subtype 2 receptor
